# Applying a meal coding system to 16-d weighed dietary record data in the Japanese context: towards the development of simple meal-based dietary assessment tools

**DOI:** 10.1017/jns.2018.21

**Published:** 2018-11-13

**Authors:** Kentaro Murakami, M. Barbara E. Livingstone, Satoshi Sasaki, Naoko Hirota, Akiko Notsu, Ayako Miura, Hidemi Todoriki, Mitsuru Fukui, Chigusa Date

**Affiliations:** 1 Department of Social and Preventive Epidemiology, School of Public Health, University of Tokyo, Tokyo, Japan; 2 Nutrition Innovation Centre for Food and Health, School of Biomedical Sciences, Ulster University, Coleraine, UK; 3 Graduate School of Health Science, Matsumoto University, Nagano, Japan; 4 Department of Food Science and Nutrition, Tottori College, Tottori, Japan; 5 Department of Health and Nutritional Sciences, Faculty of Health Promotional Sciences, Tokoha University, Shizuoka, Japan; 6 Tropical Biosphere Research Center, University of the Ryukyus, Okinawa, Japan; 7 Laboratory of Statistics, Osaka City University Medical School, Osaka, Japan; 8 Department of Food Science and Nutrition, School of Human Science and Environment, University of Hyogo, Hyogo, Japan

**Keywords:** Meals, Meal coding, Meal patterns, Food combinations, Japan, PC, principal component, PCA, principal components analysis

## Abstract

Data on the combination of foods consumed simultaneously at specific eating occasions are scarce, primarily due to a lack of assessment tools. We applied a recently developed meal coding system to multiple-day dietary intake data for assessing its ability to estimate food and nutrient intakes and characterise meal-based dietary patterns in the Japanese context. A total of 242 Japanese adults completed sixteen non-consecutive-day weighed dietary records, including 14 734 eating occasions (3788 breakfasts, 3823 lunches, 3856 dinners and 3267 snacks). Common food group combinations were identified by meal type to identify a range of generic meals. Dietary intake was calculated on the basis of not only the standard food composition database but also the substituted generic meal database. In total, eighty generic meals (twenty-three breakfasts, twenty-one lunches, twenty-four dinners and twelve snacks) were identified. The Spearman correlation coefficients between food group intakes calculated based on the standard food composition database and the substituted generic meal database ranged from 0·26 to 0·85 (median 0·69). The corresponding correlations for nutrient intakes ranged from 0·17 to 0·82 (median 0·61). A total of eleven meal patterns were established using principal components analysis, and these accounted for 39·1 % of total meal variance. Considerable variation in patterns was seen in meal type inclusion and choice of staple foods (bread, rice and noodles) and drinks, and also in meal constituents. In conclusion, this study demonstrated the usefulness of a meal coding system for assessing habitual diet, providing a scientific basis towards the development of simple meal-based dietary assessment tools.

Efforts to overcome the limitations of evaluating single nutrients and foods in isolation have led to a gradual shift in nutrition research to dietary patterns^(^^1^^,^^2^^)^. While dietary patterns are generally examined using the daily intake of individual foods^(^^3^^–^^6^^)^, an increasing number of studies now focus on dietary intake on an eating occasion basis (i.e. breakfast, lunch, dinner, and snack) or meal patterns^(^^7^^–^^9^^)^. Evaluation of meal patterns instead of overall dietary patterns might increase relevance by accounting for physiological synergies and interactions occurring during digestion and metabolism^(^^10^^)^. Moreover, meal-based dietary advice would probably be more practical as it reflects actual eating patterns^(^^9^^)^. However, data on food combinations consumed simultaneously during particular eating occasions are scarce^(^^9^^,^^11^^–^^14^^)^. Any attempt to evaluate the nearly infinite number of feasible food combinations would soon founder on the unmanageable number of individual meals. This could be overcome through the development of unique codes for identified meals^(^^12^^)^ and their subsequent use to develop inexpensive and feasible assessment tools (e.g. questionnaires)^(^^9^^)^.

The ‘frequent item sets’ data-mining method was originally developed for shopping basket analysis^(^^15^^)^. This method is designed to identify regularities in the shopping behaviour of customers and attempts to identify sets of products which are commonly purchased together^(^^15^^)^. This method might therefore be suitable for characterising combinations of foods served as a meal. We recently used this method to evaluate data from a 1-d food diary obtained from 26 361 Japanese adults in the National Health and Nutrition Survey, then developed a meal coding system and identified meal-based dietary patterns^(^^16^^)^. Analysis of the total of 94 439 meals identified ninety-four generic meals. As one example, a meal code for breakfast was built on the combination of vegetables, rice, non-alcoholic and non-energy beverages, pulses and eggs. Application of these ninety-four meal codes to principal components analysis (PCA) identified nineteen meal patterns; examples include patterns characterised by three main meals consisting of the combination of rice and vegetables.

Following this analysis, the next step was to examine whether a smaller number of generic meal codes, and thus a smaller number of meal patterns, would be identified when the meal coding system was applied to multiple-day dietary data. To test this, we applied this previous approach^(^^16^^)^ to dietary data collected using 4-d weighed dietary records in each season over a 1-year period (16 d in total). Our goal was to investigate the usefulness of the meal coding system in characterising meal-based dietary patterns and estimating food and nutrient intakes in the Japanese context, providing a scientific basis towards the development of simple generic meal-based dietary assessment tools.

## Methods

### Data source and analytic sample

The present analysis was based on data previously collected between November 2002 and September 2003 in four geographically diverse areas in Japan: Osaka (Osaka City; urban), Okinawa (Ginowan City; urban island), Nagano (Matsumoto City; rural inland) and Tottori (Kurayashi City; rural coastal). Details of that study have been provided elsewhere^(^^17^^,^^18^^)^. In brief, we sought apparently healthy women aged 30–69 years who were willing to participate and were living together with their husbands. For each area, our recruitment strategy was to include eight women for each 10-year age group (30–39, 40–49, 50–59, and 60–69 years). Their husbands were then recruited (irrespective of their age), leaving 128 people invited for each sex. Sample size was calculated to meet the major purpose of the survey, namely to validate dietary assessment questionnaires, based primarily on the size of the sample of a previous Japanese study^(^^19^^,^^20^^)^ in addition to feasibility. Inclusion criteria for this study consisted of the lack of self-report of major chronic diseases (such as diabetes and CVD) as well as community-dwelling (free-living) individuals. Excluded from the study were dietitians, those who had experienced dietary counselling from a doctor or dietitian, and those who had a history of hospitalisation for diabetes education. Group meetings were held prior to the study to explain the purpose and protocol of the study, at which time written informed consent was obtained from all participants. In total, 121 women and 121 men completed the survey protocol and were included in the analysis.

This survey was conducted according to the guidelines laid down in the Declaration of Helsinki. The use of the study data was approved by the University of Tokyo Faculty of Medicine Ethics Committee.

### Dietary assessment

Data on dietary intakes were obtained using 4 × 4 d (total 16 d) weighed dietary records, as described previously^(^^17^^)^. Briefly, the participants were requested to maintain a dietary record over four non-consecutive days once per season at an interval of about 3 months, namely in November and December 2002 for autumn, February 2003 for winter, May 2003 for spring, and August and September 2003 for summer. The sets of four recording days each comprised one weekend day and three weekdays within approximately 2 weeks. In the orientations, locally employed registered dietitians provided the participants with written and verbal directions on maintaining the dietary record, and provided them with a sample completed record as an example. The couples were each provided with recording sheets and a KD-173 digital scale (Tanita; precision ± 2 g at 0–250 g and ± 4 g at 251–1000 g) and instructed on how each food and drink should be weighed. They were requested to document and weigh all foods and drinks taken on each of the recording days. On occasions when weighing was problematic (e.g. dining out), they were instructed to document as much information as possible, including the brand name of the food, the consumed portion size (based on typical household measures), as well as the details of leftovers. The participants were asked to fax the completed forms for each recording day to the local staff, who then reviewed the forms and, whenever necessary, sought additional information or modification of the record via telephone or fax. The responses were generally faxed to the centre, although some were handed directly to the centre staff. The collected records were then all reviewed by trained registered dietitians at each local centre, and again at in the study centre. As requested in the study protocol, portion sizes estimated using household measures were converted into weights, and individual food items were coded based on the Standard Tables of Food Composition in Japan^(^^21^^)^. This food composition table is a complete and widely used one in Japan, containing nutrient content information for >1800 food items.

### Development of the meal coding system

#### Classification of breakfast, lunch, dinner and snacks

The food diary sheet was designed to accord with a typical Japanese eating pattern, namely breakfast, lunch, dinner and snacks. Because these eating occasions were described accordingly in the diary, the meal types we used in the analysis were based on this classification. A total of 14 734 eating occasions (3788 breakfasts, 3823 lunches, 3856 dinners and 3267 snacks) were identified from the original 206 837 food item entries.

#### Definition of food groups

In accordance with our previous study^(^^16^^)^, each unique food item eaten by the participants (>1300 individual food item codes) was reclassified and coded into one of twenty specified food groups. Three food groups (sugars, fats and oils, and seasonings) usually consumed with other foods were excluded^(^^16^^)^, and the analysis utilised the remaining seventeen groups (Supplementary Table S1). Grouping of foods was done based on similarities in nutrient profile or culinary use of the foods, mainly in accordance with the Standard Tables of Food Composition in Japan^(^^21^^)^ and the National Health and Nutrition Survey's classification of food groups^(^^22^^)^.

#### Meal coding procedure based on food group combinations

With all meal types (breakfast, lunch, dinner and snacks), we initially evaluated common food group combinations for individual meals^(^^16^^)^. The individual meals were then coded as generic meals. Preliminary scrutiny based on food groups having consumption >0 g within a meal was unable to adequately categorise the various food group combinations due to the inability to distinguish major and minor constituents within a meal. We therefore limited consideration to those food groups having consumption of >15 g within a meal, as determined arbitrarily based on the size of a measuring cup (15 ml) commonly used in Japan for seasonings or toppings^(^^16^^)^. We categorised the most commonly consumed combinations via a similar process to ‘frequent item sets’, an *a priori* methodology used in data mining^(^^11^^,^^15^^,^^16^^)^. For example, for the breakfast meal type (Supplementary Table S2; *n* 3788), the most commonly consumed combination of food groups at >15 g was ‘vegetables and tea and coffee’. Among individual breakfasts comprising a combination of ‘vegetables and tea and coffee’ (*n* 1930), the most commonly consumed food group was ‘rice’. For all individual breakfasts consisting of the ‘vegetables, tea and coffee and rice’ combination (*n* 1362), the most commonly consumed food group was ‘pulses’. For individual breakfasts consisting of the ‘vegetables, tea and coffee, rice and pulses’ combination (*n* 669), the most frequently consumed food group was ‘fruit’. For all individual breakfasts consisting of the ‘vegetables, tea and coffee, rice, pulses and fruit’ combination (*n* 258), the most commonly consumed food group was ‘dairy products’. Based on this, we considered that the ‘vegetables, tea and coffee, rice, pulses, fruit and dairy products’ group (*n* 129) represented a food group combination pattern and labelled it with the generic meal code of 1101. We arbitrarily determined the number of included food groups based on the frequency of meals (breakfasts in this case) included in the code, with consideration to the importance of food groups in characterising the code, and also our goal to restrict the total number of generic meals to eighty or fewer. After categorising breakfasts which included combinations of ‘vegetables and tea and coffee’, we identified the next most commonly consumed food group combination (‘bread and dairy products’) and categorised it similarly. We then repeated this process stepwise until arriving at the point at which the next most commonly consumed food group combination represented <2 % of total breakfast number (in this case, ‘dairy products and tea and coffee’). We then identified breakfasts which consisted of not only a single food group, but which also accounted for >2 % of the number of total breakfasts, and identified these as one category (in this case, ‘tea and coffee’). We repeated this process stepwise for the other meal types. Finally, we established eighty generic meals accounting for all meal types. Each meal was then assigned a unique generic meal code (Supplementary Table S2 for breakfast; and Supplementary Tables S3–S5 for lunch, dinner and snacks, respectively).

#### Estimation of nutrient contents of generic meals

In accordance with our previous study^(^^16^^)^, we estimated the nutrient content of each coded generic meal using the aggregation of the nutrient composition of individual meals assigned to that code, as follows. First, for each meal for each individual we calculated the total amount (g), weight of each food group (shown in Supplementary Table S1), and content of each nutrient, using the Standard Tables of Food Composition in Japan^(^^21^^)^. Then, we calculated mean values of these variables based on all meals classified for each meal code. A compilation of these (mean) values was thus the generic meal database used in this analysis.

### Statistical analysis

#### Comparisons of intakes of energy, food groups and nutrients between the original food composition database and the generic meal database

All statistical analyses were performed using SAS statistical software (version 9.4; SAS Institute Inc.). Daily intakes of energy, food groups and nutrients were calculated using the Standard Tables of Food Composition in Japan^(^^21^^)^ and also using data in the substituted generic meal database. For all dietary variables, mean daily values over 16 d were used in the analysis. Descriptive data are presented as means and standard deviations, except for food group intakes from each meal, for which medians and 25th and 75th percentiles are used (due to highly skewed data). Food group and nutrient intakes are expressed as both crude values (amount per d) and energy-adjusted values based on the density method (i.e. % of energy for energy-providing nutrients and amount per 4184 kJ (1000 kcal) of energy for food groups and other nutrients). The association between the intake of each of energy, food groups and nutrients within the two databases was assessed using Spearman's correlation coefficient.

#### Determination of meal-based dietary patterns

Meal-based dietary patterns were evaluated using PCA based on the percentage contribution of daily energy intake for each of the eighty generic meals eaten by each participant (based on the generic meal database). The number of retained components was evaluated by examining the scree plot along with the combination of meals on the principal components (PC) identified^(^^23^^–^^25^^)^. Generic meals having an absolute loading value >0·25 were held to have contributed to the component, and cross-loading of components was permitted^(^^6^^,^^25^^,^^26^^)^. Presented data are derived from the Varimax rotation.

## Results

The present analysis included 242 Japanese adults (121 women aged 31–69 years and 121 men aged 31–81 years) with a mean age of 51·0 years (Table 1). For breakfast, lunch and snacks, the median of tea and coffee consumed was the largest among the seventeen food groups considered in the development of the generic meal database (Table 2). The next most commonly consumed food groups, however, differed considerably, namely rice, vegetables, dairy products, fruit and bread for breakfast; rice, vegetables, noodles, fish and meat for lunch; and confectioneries and dairy products for snacks. For dinner, vegetables were the most commonly consumed food group, followed by tea and coffee, rice, fish, meat, pulses and potatoes.

**Table 1.** Basic characteristics of participants (Mean values and standard deviations)

	Women (*n* 121)		Men (*n* 121)		Total (*n* 242)	
	Mean	sd	Mean	sd	Mean	sd
Age (years)	49·6	11·3	52·4	512·2	51·0	11·8
Body height (m)	1·55	0·06	1·67	0·07	1·61	0·09
Body weight (kg)	53·3	7·1	66·4	10·4	59·9	11·0
BMI (kg/m^2^)	22·3	2·8	23·6	2·9	23·0	2·9

**Table 2.** Food group intake (g/d) from each meal (*n* 242)* (Medians and 25th and 75th percentiles)

	Breakfast			Lunch			Dinner			Snack		
	Median	25th percentile	75th percentile	Median	25th percentile	75th percentile	Median	25th percentile	75th percentile	Median	25th percentile	75th percentile
Rice	70·9	8·8	127·7	125·4	90·9	166·9	124·6	92·5	162·5	0	0	0
Bread	18·0	2·3	41·9	1·3	0	9·4	0	0	0·3	0	0	2·1
Noodles	0	0	0	39·1	16·3	68·6	18·1	6·3	34·0	0	0	0
Other grains	1·3	0	4·7	6·3	3·0	11·2	9·0	4·5	13·5	0	0	2·1
Potatoes	3·1	0	9·2	9·2	4·3	17·1	21·4	14·2	30·2	0	0	3·2
Pulses†	9·4	0·9	22·7	10·7	4·6	20·4	27·3	18·8	40·9	1·1	0	4·2
Vegetables	53·5	17·9	89·0	85·2	57·3	112·3	141·0	112·0	174·0	0·1	0	4·3
Fruit	21·3	6·4	44·9	15·1	5·0	33·1	19·0	7·7	39·5	12·4	0·8	32·5
Fish‡	5·6	0·7	16·5	23·7	14·8	34·1	48·0	34·8	64·7	0	0	0·6
Meat	4·6	1·0	8·8	20·6	14·1	31·3	38·2	25·7	54·9	0	0	0
Eggs	14·0	5·4	24·7	12·2	8·1	17·7	8·7	5·6	11·9	0	0	0·6
Dairy products	49·9	18·1	118·9	6·2	1·0	19·4	6·2	1·0	19·7	22·2	7·3	52·0
Confectioneries	0	0	4·6	0	0	6·3	0	0	4·4	26·5	11·3	42·1
Fruit and vegetable juice	0	0	0	0	0	0	0	0	0	0	0	0
Alcoholic beverages	0	0	0·1	0·6	0·1	1·9	36·8	2·2	203·8	0	0	24·2
Soft drinks	0	0	6·3	0	0	5·2	0	0	0	12·7	0	40·5
Tea and coffee	145·7	102·9	217·6	154·2	117·1	202·7	126·4	61·9	187·5	238·1	120·3	371·0
Sugar§	2·1	0·7	4·6	1·9	1·1	2·9	2·6	1·5	3·6	1·1	0·2	3·1
Fats and oils§	2·2	1·0	3·7	4·8	3·2	6·5	5·4	3·6	7·3	0	0	0·6
Seasonings§	20·5	5·8	67·6	48·4	32·4	77·7	63·6	40·2	97·7	0·1	0	1·3

* Calculated based on the original food composition database.

† Including nuts.

‡ Including shellfish.

§ Not considered in the development of the substituted generic meal database.

### Identified generic meals

In total, eighty generic meals were identified: twenty-three for breakfast, twenty-one for lunch, twenty-four for dinner and twelve for snacks (Supplementary Tables S2–S5, respectively). For breakfast, the most frequently identified food group combination was ‘vegetables and tea and coffee’ (twelve meals), *v.* ‘rice and vegetables’ for lunch (fourteen meals) and dinner (eighteen meals). However, because the most frequent accompaniment among meals that consisted of these combinations was rice for breakfast and tea and coffee for lunch and dinner, the combination ‘rice, vegetables, and tea and coffee’ was most frequently identified for all three main meals (eight meals for breakfast, ten meals for lunch and eleven meals for dinner). Pulses were the next most frequent accompaniment for breakfast, whereas the corresponding food groups for lunch and dinner were fish and meat. The next most frequently identified food group combinations differed considerably, with ‘bread and dairy products’ for breakfast (four meals), ‘noodles and tea and coffee’ for lunch (three meals) and ‘vegetables and meat’ for dinner (three meals). The most prevalent combination for snacks was ‘confectioneries and tea and coffee’ (three meals), followed by ‘dairy products and tea and coffee’ (one meal) and ‘fruit and tea and coffee’ (one meal). Snacks consisting of one of the food groups only were also common.

### Comparison of intakes of energy, food groups and nutrients between the two databases

Dietary intakes were calculated on the basis of not only the standard food composition database but also the substituted generic meal database. As an artefact of the procedure used to develop the generic meal database, mean values of energy intake (Table 3) and crude intakes of food groups (Supplementary Table S6) and nutrients (Supplementary Table S7) were (theoretically) identical for both calculations and thus those of energy-adjusted intakes of food groups (Table 3) and nutrients (Table 4) were closely similar. In contrast, standard deviation values were smaller when the generic meals database was used. The Spearman correlation coefficient for energy intake was 0·47. In terms of energy-adjusted intakes, the correlations ranged from 0·26 to 0·85 (median 0·69) for the seventeen food groups and from 0·17 to 0·82 (median 0·61) for the forty-two nutrients examined. Similar correlations were observed for crude intakes of food groups (median 0·70; range 0·30–0·83) and nutrients (median 0·59; range 0·26–0·81).

**Table 3.** Daily energy intake and energy-adjusted food group intakes (g/4184 kJ) calculated using a standard food composition database and the substituted generic meal database and correlations between intakes calculated using the two databases (*n* 242)* (Mean values and standard deviations and Spearman's correlation coefficients)

	Standard food composition database		Generic meal database		*P* (paired *t* test)	Spearman's *r*
	Mean	sd	Mean	sd
Energy (kJ/d)	8820	1866	8820	753	1·00	0·47
Rice	157·6	44·7	158·4	32·2	0·64	0·77
Bread	17·2	13·2	16·9	11·0	0·56	0·85
Noodles	35·0	20·6	34·8	15·4	0·92	0·64
Other grains	12·4	9·9	12·5	3·1	0·89	0·26
Potatoes	20·9	9·9	20·5	3·3	0·49	0·45
Pulses†	30·9	17·0	30·2	6·9	0·38	0·67
Vegetables	142·3	49·0	139·3	16·2	0·24	0·64
Fruit	50·2	35·3	48·9	10·4	0·48	0·69
Fish‡	42·5	16·4	42·3	9·7	0·77	0·78
Meat	35·0	14·5	35·1	8·4	0·89	0·81
Eggs	19·3	7·6	19·0	3·7	0·38	0·55
Dairy products	68·0	46·7	66·6	21·9	0·55	0·74
Confectioneries	17·3	11·1	16·8	7·3	0·29	0·76
Fruit and vegetable juice	6·6	14·2	6·6	2·7	0·94	0·30
Alcoholic beverages	77·4	109·2	84·8	43·1	0·17	0·81
Soft drinks	23·0	28·6	23·6	11·5	0·67	0·60
Tea and coffee	343·7	153·5	334·7	81·3	0·23	0·72
Sugar§	5·2	4·2	5·2	1·1	0·93	0·42
Fats and oils§	6·6	2·5	6·6	0·7	0·86	0·58
Seasonings§	84·8	41·6	83·0	10·8	0·48	0·15

* As a result of the procedure used to develop the generic meal database, mean values of energy intake (and crude food group and nutrient intakes) for the two calculations were theoretically identical.

† Including nuts.

‡ Including shellfish.

§ Not considered in the development of the substituted generic meal database.

**Table 4.** Energy-adjusted nutrient intakes calculated using a standard food composition database and the substituted generic meal database and correlations between intakes calculated using the two databases (*n* 242)* (Mean values and standard deviations and Spearman's correlation coefficients)

	Standard food composition database		Generic meal database		*P* (paired *t* test)	Spearman's *r*
	Mean	sd	Mean	sd
Protein (% energy)	14·8	1·7	14·7	0·5	0·24	0·59
Total fat (% energy)	26·7	3·9	26·6	1·4	0·43	0·61
SFA (% energy)	7·5	1·5	7·4	0·6	0·50	0·68
MUFA (% energy)	9·5	1·8	9·5	0·6	0·63	0·65
PUFA (% energy)	6·1	0·9	6·0	0·2	0·35	0·28
*n*-6 PUFA (% energy)	4·9	0·8	4·9	0·2	0·35	0·41
*n*-3 PUFA (% energy)	1·2	0·2	1·1	0·1	0·64	0·47
Marine-origin *n*-3 PUFA (% energy)†	0·4	0·2	0·4	0·1	0·94	0·68
EPA (% energy)	0·14	0·06	0·14	0·03	0·95	0·68
DHA (% energy)	0·24	0·10	0·24	0·05	0·92	0·67
α-Linolenic acid (% energy)	0·67	0·12	0·66	0·03	0·50	0·17
Carbohydrate (% energy)	53·9	5·3	53·7	2·3	0·53	0·69
Alcohol (% energy)	3·5	4·8	4·0	2·0	0·08	0·82
Cholesterol (mg/4184 kJ)	174·3	37·7	172·3	15·7	0·37	0·47
Total dietary fibre (g/4184 kJ)	7·4	2·0	7·3	0·6	0·22	0·78
Soluble dietary fibre (g/4184 kJ)	1·6	0·4	1·6	0·1	0·18	0·70
Insoluble dietary fibre (g/4184 kJ)	5·3	1·4	5·2	0·4	0·27	0·80
Retinol (μg/1000 kcal (4184 kJ))	186·1	202·1	182·2	27·8	0·76	0·19
Vitamin A (retinol equivalents) (μg/4184 kJ)‡	342·2	218·3	333·8	27·5	0·54	0·24
α-Carotene (μg/4184 kJ)	242·8	136·8	235·1	31·8	0·36	0·33
β-Carotene (μg/4184 kJ))	1618·0	643·6	1571·0	192·6	0·20	0·49
β-Carotene equivalents (μg/4184 kJ)§	1859·0	743·6	1806·0	210·0	0·21	0·50
Cryptoxanthin (μg/4184 kJ)	171·3	155·5	165·7	39·2	0·54	0·46
α-Tocopherol (mg/4184 kJ)	3·8	0·7	3·8	0·2	0·32	0·48
Vitamin K (μg/4184 kJ)	119·4	50·3	117·0	17·9	0·36	0·62
Thiamin (mg/4184 kJ)	0·47	0·07	0·46	0·02	0·43	0·19
Riboflavin (mg/4184 kJ)	0·68	0·13	0·67	0·04	0·24	0·62
Niacin (mg/4184 kJ)	9·4	1·9	9·4	0·5	0·72	0·40
Vitamin B_6_ (mg/4184 kJ)	0·66	0·13	0·66	0·03	0·52	0·53
Vitamin B_12_ (μg/4184 kJ)	4·1	1·6	4·1	0·6	0·73	0·61
Folate (μg/4184 kJ)	183·9	52·0	181·0	13·2	0·32	0·63
Pantothenic acid (mg/4184 kJ)	3·1	0·5	3·1	0·1	0·23	0·59
Vitamin C (mg/4184 kJ)	56·4	21·2	55·2	5·2	0·32	0·69
Na (mg/4184 kJ)	2471·0	840·9	2449·0	176·0	0·68	0·17
K (mg/4184 kJ)	1367·0	282·4	1347·0	74·0	0·19	0·71
Ca (mg/4184 kJ)	281·1	79·6	276·5	29·2	0·24	0·77
Mg (mg/4184 kJ)	145·0	30·9	143·2	8·5	0·30	0·67
P (mg/4184 kJ)	558·1	78·6	553·1	24·5	0·23	0·70
Fe (mg/4184 kJ)	4·3	0·9	4·2	0·3	0·23	0·58
Zn (mg/4184 kJ)	4·3	0·5	4·3	0·2	0·40	0·41
Cu (mg/4184 kJ)	0·61	0·10	0·61	0·04	0·34	0·71
Mn (mg/4184 kJ)	2·0	1·0	2·0	0·2	0·77	0·67

* As a result of the procedure used to develop the generic meal database, mean values of energy intake as well as crude nutrient and food group intakes for the two calculations were theoretically identical.

† Sum of EPA, DPA and DHA.

‡ Sum of retinol, 1/12 of β-carotene, 1/24 of α-carotene and 1/24 of cryptoxanthin.

§ Sum of β-carotene, 1/2 of α-carotene and 1/2 of cryptoxanthin.

### Meal-based dietary patterns

PCA identified eleven PC or meal patterns, which together accounted for 39·1 % of total variance (Table 5 for PC 1–5 and Supplementary Table S8 for PC 6–11). PC 1 was characterised by three main meals consisting of the combination of rice and vegetables (without tea and coffee), as well as meat, fish or both during lunch and dinner. PC 2 was characterised by breakfast and lunch consisting of rice, vegetables and tea and coffee, in addition to confectioneries and dairy products as snacks. PC 3 was characterised by dinner consisting of vegetables, meat, fish and alcoholic beverages. PC 4 was characterised by breakfast consisting of tea and coffee only or the combination of bread and tea and coffee, as well as lunch, dinner and snacks consisting of all other combinations. PC 5 was characterised by breakfast consisting of bread, dairy products and tea and coffee, and lunch and dinner consisting of rice, vegetables, tea and coffee, fish and pulses. PC 6 was characterised by breakfast consisting of the combination of bread and dairy products or all other combinations, as well as snacks consisting of confectioneries only or all other combinations. PC 7 was characterised by breakfast and dinner consisting of rice, vegetables and tea and coffee, as well as lunch consisting of noodles, vegetables and tea and coffee. PC 8 was characterised by breakfast and dinner consisting of rice, vegetables and tea and coffee; lunch consisting of the combination of vegetables and meat, of bread and dairy products or of all other combinations; and snacks consisting of confectioneries and tea and coffee. PC 9 had a similar pattern to PC 5, except for fish and pulses. PC 10 was characterised by breakfast consisting of rice and tea and coffee (without vegetables); lunch consisting of rice, vegetables, fish, meat, eggs and tea and coffee; and snacks consisting of soft drinks, tea and coffee or both. PC 11 was characterised by breakfast consisting of vegetables and tea and coffee (without rice or bread); lunch consisting of rice, vegetables, fish, eggs and non-alcoholic and non-energy beverages; and dinner consisting of vegetables and meat (without rice).

**Table 5.** Principal components analysis (PCA) of meals based on eighty generic meal codes, showing the dominant loading values for each principal component (PC) (1–5)*

PC (variance)	Meal code	Meal type	Meal description	Loading value
1 (5·6 %)	1301	Breakfast	Rice, vegetables, dairy products (without tea and coffee, bread)	0·53
	1302	Breakfast	Rice, vegetables (without tea and coffee, dairy products, alcoholic beverages)	0·51
	2105	Lunch	Rice, vegetables, tea and coffee, fish (without meat, eggs, pulses)	−0·27
	2108	Lunch	Rice, vegetables, tea and coffee, meat (without fish, eggs, potatoes)	−0·30
	2111	Lunch	Rice, vegetables, meat, fish (without tea and coffee, bread)	0·50
	2112	Lunch	Rice, vegetables, meat (without tea and coffee, fish)	0·47
	2113	Lunch	Rice, vegetables, fish (without tea and coffee, meat)	0·56
	2114	Lunch	Rice, vegetables (without tea and coffee, meat, fish, fruit and vegetable juice)	0·52
	2203	Lunch	Noodles, tea and coffee (without vegetables, bread, fruit and vegetable juice)	−0·31
	3105	Dinner	Rice, vegetables, tea and coffee, fish, meat (without pulses, potatoes)	−0·26
	3107	Dinner	Rice, vegetables, tea and coffee, fish (without pulses, meat, fruit)	−0·29
	3108	Dinner	Rice, vegetables, tea and coffee, meat, potatoes (without fish, soft drinks)	−0·28
	3109	Dinner	Rice, vegetables, tea and coffee, meat, pulses (without fish, potatoes)	−0·27
	3114	Dinner	Rice, vegetables, fish, pulses (without tea and coffee, alcoholic beverages)	0·63
	3115	Dinner	Rice, vegetables, fish (without tea and coffee, pulses, alcoholic beverages)	0·63
	3116	Dinner	Rice, vegetables, meat, potatoes (without tea and coffee, fish)	0·45
	3117	Dinner	Rice, vegetables, meat (without tea and coffee, fish, potatoes, bread)	0·43
	3118	Dinner	Rice, vegetables (without tea and coffee, fish, meat, soft drinks)	0·37
2 (3·9 %)	1102	Breakfast	Vegetables, tea and coffee, rice, pulses, fruit (without dairy products)	0·29
	1103	Breakfast	Vegetables, tea and coffee, rice, pulses, eggs (without fruit, fruit and vegetable juice, alcoholic beverages)	0·41
	1104	Breakfast	Vegetables, tea and coffee, rice, pulses, fish (without fruit, eggs, fruit and vegetable juice, alcoholic beverages, bread, noodles)	0·44
	1106	Breakfast	Vegetables, tea and coffee, rice, eggs (without pulses)	0·36
	1107	Breakfast	Vegetables, tea and coffee, rice, fish (without pulses, eggs)	0·41
	1108	Breakfast	Vegetables, tea and coffee, rice (without pulses, eggs, fish, alcoholic beverages)	0·44
	1201	Breakfast	Bread, dairy products, tea and coffee, fruit (without vegetables, noodles, alcoholic beverages)	−0·32
	1202	Breakfast	Bread, dairy products, tea and coffee (without fruit, vegetables, noodles, alcoholic beverages)	−0·62
	1601	Breakfast	Dairy products, tea and coffee (without vegetables, rice, bread, noodles, alcoholic beverages)	−0·44
	2104	Lunch	Rice, vegetables, tea and coffee, fish, pulses (without meat, eggs, bread, soft drinks)	0·27
	2105	Lunch	Rice, vegetables, tea and coffee, fish (without meat, eggs, pulses)	0·47
	2109	Lunch	Rice, vegetables, tea and coffee, pulses (without fish, meat, bread)	0·29
	2203	Lunch	Noodles, tea and coffee (without vegetables, bread, fruit and vegetable juice)	−0·42
	4101	Snack	Confectioneries, tea and coffee, dairy products	−0·28
	4201	Snack	Dairy products, tea and coffee (without confectioneries)	−0·39
	4501	Snack	Confectioneries, dairy products (without tea and coffee, noodles, fish)	0·28
3 (3·8 %)	2110	Lunch	Rice, vegetables, tea and coffee (without fish, meat, pulses)	−0·31
	3107	Dinner	Rice, vegetables, tea and coffee, fish (without pulses, meat, fruit)	−0·37
	3108	Dinner	Rice, vegetables, tea and coffee, meat, potatoes (without fish, soft drinks)	−0·38
	3109	Dinner	Rice, vegetables, tea and coffee, meat, pulses (without fish, potatoes)	−0·27
	3110	Dinner	Rice, vegetables, tea and coffee, meat (without fish, pulses, potatoes)	−0·42
	3111	Dinner	Rice, vegetables, tea and coffee (without fish, meat, bread)	−0·34
	3113	Dinner	Rice, vegetables, fish, alcoholic beverages (without tea and coffee, meat)	0·37
	3201	Dinner	Vegetables, meat, alcoholic beverages, fish (without rice, fruit and vegetable juice)	0·69
	3202	Dinner	Vegetables, meat, alcoholic beverages (without rice, fish)	0·70
	3301	Dinner	Vegetables, fish (without rice, meat)	0·57
	3401	Dinner	Rice, tea and coffee (without vegetables)	−0·33
4 (3·5 %)	1501	Breakfast	Bread, tea and coffee (without vegetables, dairy products, rice, noodles, alcoholic beverages)	0·40
	1701	Breakfast	Tea and coffee only	0·59
	2106	Lunch	Rice, vegetables, tea and coffee, meat, eggs (without fish, alcoholic beverages)	−0·29
	2203	Lunch	Noodles, tea and coffee (without vegetables, bread, fruit and vegetable juice)	0·46
	2401	Lunch	Rice, tea and coffee (without vegetables, noodles, alcoholic beverages)	0·40
	2601	Lunch	All other combinations	0·54
	3117	Dinner	Rice, vegetables, meat (without tea and coffee, fish, potatoes, bread)	0·39
	3501	Dinner	All other combinations	0·39
	4101	Snack	Confectioneries, tea and coffee, dairy products	−0·33
	4102	Snack	Confectioneries, tea and coffee, fruit (without dairy products)	−0·31
	4103	Snack	Confectioneries, tea and coffee (without dairy products, fruit)	−0·30
	4201	Snack	Dairy products, tea and coffee (without confectioneries)	−0·27
	4801	Snack	All other combinations	0·42
5 (3·3 %)	1107	Breakfast	Vegetables, tea and coffee, rice, fish (without pulses, eggs)	−0·25
	1109	Breakfast	Vegetables, tea and coffee, bread, dairy products, eggs (without rice, noodles, alcoholic beverages)	0·41
	1201	Breakfast	Bread, dairy products, tea and coffee, fruit (without vegetables, noodles, alcoholic beverages)	0·55
	2104	Lunch	Rice, vegetables, tea and coffee, fish, pulses (without meat, eggs, bread, soft drinks)	0·51
	2108	Lunch	Rice, vegetables, tea and coffee, meat (without fish, eggs, potatoes)	−0·25
	3101	Dinner	Rice, vegetables, tea and coffee, fish, pulses, meat	0·31
	3102	Dinner	Rice, vegetables, tea and coffee, fish, pulses, fruit (without meat, soft drinks)	0·50
	3103	Dinner	Rice, vegetables, tea and coffee, fish, pulses (without meat, fruit)	0·51
	3110	Dinner	Rice, vegetables, tea and coffee, meat (without fish, pulses, potatoes)	−0·41
	3401	Dinner	Rice, tea and coffee (without vegetables)	−0·34
	3501	Dinner	All other combinations	−0·31

* Loading values were calculated from PCA using the percentage contribution of daily energy intake of the eighty generic meals for each participant (*n* 242). Only loading values <–0·25 or >0·25 are displayed. The results for other PC (6–11) are shown in Supplementary Table S8.

## Discussion

Here, we applied a previously established meal coding system^(^^16^^)^ to data obtained from 16-d dietary records from 242 Japanese adults, and demonstrated the usefulness of a meal coding system for assessing habitual diet. The present study thus provided a scientific basis towards the development of simple generic meal-based dietary assessment tools.

Our motivation in developing this meal coding system was to reduce the complexity involved in dealing with multiple food combinations across all meals. The seventeen food groups in four meal types in this study could produce a theoretical figure of approximately 3·0 × 10^20^ meal combinations. In contrast, use of only eighty generic meals decreases this number markedly, to about 1·4 × 10^5^ combinations. The Spearman correlation between energy intake calculated based on the standard food composition database and that calculated using the substituted generic meal database was moderate. Correlations for thirteen (76 %) of the seventeen food groups used in the development of the generic meal database (i.e. rice, bread, noodles, pulses, vegetables, fruit, fish, meat, dairy products, confectioneries, alcoholic beverages, soft drinks and tea and coffee) were relatively high (≥0·60), a not unexpected result given speculation that these foods are consumed in relatively fixed amounts in each eating occasion in Japan. The low correlations seen for the four other food groups (other grains, potatoes, eggs and fruit and vegetable juice) as well as food groups not considered in the development of the database (sugar, fats and oils and seasonings) are again not unexpected, because these were consumed in very limited or varying amounts on each occasion, and tend to be invisible or unquantifiable within meals. Reflecting the correlations for food groups, those for more than half of the nutrients examined (22 of 42) were relatively high (≥0·60). Compared with the standard food composition table, the substituted generic meal database was therefore reasonably suitable for ranking individuals, for a number of dietary variables at least. This does not nevertheless provide sufficient evidence to validate this specific generic meal database, being completely dependent on the original calculation, and thus inevitably overestimating the correlations. It would be interesting to determine whether correlations are still sufficiently high when the generic meal database is applied to other dietary intake data obtained independently.

Generally speaking, most of the food group combinations, generic meals and meal-based dietary patterns that we identified here were also observed in our previous analysis conducted using data from the National Health and Nutrition Survey^(^^16^^)^. For example, in both the present and previous studies, we observed patterns characterised by three main meals which consisted of the combination of rice and vegetables (PC 1 in this study and PC 2, 3, 5 and 6 in the previous study); by the consumption of alcoholic beverages at dinner (PC 3 in this study and PC 17 in the previous study); by meals mainly consisting of all other combinations (PC 4 in this study and PC 1 in the previous study); and by breakfast and dinner consisting of the combination of rice and vegetable as well as lunch without rice (PC 7 and 8 in this study and PC 10 in the previous study). Nevertheless, the number of both generic meals and meal-based dietary patterns identified in this study based on 16-d dietary data was smaller than that based on 1-d data (80 *v.* 94 for generic meals; 11 *v.* 19 for meal-based dietary patterns). This is expected, given that meal patterns derived from 1-d dietary data are unlikely to represent the usual dietary pattern of the individual respondents, while the use of 16-d dietary data would reduce the variability of meals. Moreover, the eleven meal patterns identified here accounted for 39·1 % of total variance, which is high compared with previous meal pattern analyses in Japan (24·1 %)^(^^16^^)^ and Ireland (29·3 % by twelve patterns)^(^^11^^)^, as well as with many analyses of overall dietary pattern^(^^3^^,^^4^^,^^6^^,^^23^^–^^26^^)^. In any case, this study provides basic information for future investigation of food combinations in meals.

Meal patterns can be analysed and categorised in a number of different ways. For example, meal patterns can be derived from PCA or cluster analysis as well as latent class analysis^(^^27^^)^ based on food group intakes from each eating occasion^(^^13^^)^, albeit that this requires detailed information on dietary intake (usually from dietary record or 24-h recall). While additional work is required to accurately assess meal-based dietary patterns for individuals, the meal coding system developed here, which was based on information on habitual dietary intake, aids in developing inexpensive and feasible assessment tools for meal patterns (e.g. paper- or web-based questionnaires).

Several limitations of the present study warrant mention. First, the generalisability of the findings might be limited because the participants were not a representative sample of general Japanese but rather volunteers, and thus possibly health conscious. Nevertheless, it should be noted that most of the food group combinations and the generic meals identified were similar to those derived from the National Health and Nutrition Survey^(^^16^^)^, as mentioned above. Second, all self-reported dietary assessment methods are subject to both random and systematic measurement errors^(^^28^^)^. To minimise these, we evaluated dietary habits using 4-d weighed dietary records collected in each of the four seasons over a 1-year period, although dietary records are also susceptible to measurement error due to erroneous recording and potential changes in eating behaviour^(^^29^^)^. Finally, although generic meal classification methods are often considered to be subjective, this subjectivity is commonly encountered in dietary estimation studies^(^^11^^,^^12^^)^. Subjectivity may also occur in the way individual participants define meal types, and a standardised protocol for meal-type designation is not currently available^(^^9^^,^^30^^,^^31^^)^. Further, PCA itself is subject to a number of limitations, and may produce data-specific results. The present study was further limited by analytic decisions which were themselves arbitrary or subjective at a number of points, such as the number and classification of meal codes (and thus of food groups); the form of meal input variables and adjustment for energy intake (i.e. % of energy); the number of extracted components and the rotation method used; and also the interpretation of components. In total, our process might have produced some degree of inconsistency, and the results and process used to derive the meal patterns accordingly warrant cautious interpretation. Numerous other factors associated with overall dietary patterns, in some populations at least, such as sex and age^(^^3^^)^, may make an impact on generic meal code types and the meal-based patterns derived therefrom. Although beyond the scope of the present study, these might be examined in future analysis. Nevertheless, the eleven meal patterns identified here were based on information on habitual diets (assessed by 4-d dietary records in each season over a 1-year period) and represent a considerable advance in the meal-based approach. Further, they provide important insights into novel concepts of food combination for future nutrition research.

In conclusion, this study based on 16-d weighed dietary record data demonstrated the usefulness of a meal coding system for investigating the complex nature of Japanese meals and estimating intakes of selected foods and nutrients. The next step is to use these results to develop and validate inexpensive and feasible assessment tools to assess meal or food combination patterns (e.g. paper- or web-based questionnaires) by combination of the generic meal database identified here. This would make it possible to conduct large-scale epidemiological research on meal or food combination patterns in relation to diet quality and health status.

## Supplementary material

For supplementary material accompanying this paper visit https://doi.org/10.1017/jns.2018.21. click here to view supplementary material

## References

[1] Kant AK (2004) Dietary patterns and health outcomes. J Am Diet Assoc 104, 615–635. 15054348 10.1016/j.jada.2004.01.010

[2] Hu FB (2002) Dietary pattern analysis: a new direction in nutritional epidemiology. Curr Opin Lipidol 13, 3–9. 11790957 10.1097/00041433-200202000-00002

[3] Newby PK & Tucker KL (2004) Empirically derived eating patterns using factor or cluster analysis: a review. Nutr Rev 62, 177–203. 15212319 10.1301/nr.2004.may.177-203

[4] Ax E, Warensjö Lemming E, Becker W, (2016) Dietary patterns in Swedish adults; results from a national dietary survey. Br J Nutr 115, 95–104. 26490112 10.1017/S0007114515004110

[5] Gazan R, Bechaux C, Crepet A, (2016) Dietary patterns in the French adult population: a study from the second French national cross-sectional dietary survey (INCA2) (2006–2007). Br J Nutr 116, 300–315. 27189191 10.1017/S0007114516001549 PMC4910537

[6] Hearty AP & Gibney MJ (2009) Comparison of cluster and principal component analysis techniques to derive dietary patterns in Irish adults. Br J Nutr 101, 598–608. 18577300 10.1017/S0007114508014128

[7] Kerver JM, Yang EJ, Obayashi S, (2006) Meal and snack patterns are associated with dietary intake of energy and nutrients in US adults. J Am Diet Assoc 106, 46–53. 16390666 10.1016/j.jada.2005.09.045

[8] Myhre JB, Loken EB, Wandel M, (2015) Meal types as sources for intakes of fruits, vegetables, fish and whole grains among Norwegian adults. Public Health Nutr 18, 2011–2021. 25384694 10.1017/S1368980014002481 PMC10271256

[9] Leech RM, Worsley A, Timperio A, (2015) Understanding meal patterns: definitions, methodology and impact on nutrient intake and diet quality. Nutr Res Rev 28, 1–21. 25790334 10.1017/S0954422414000262 PMC4501369

[10] Jacobs DR Jr & Steffen LM (2003) Nutrients, foods, and dietary patterns as exposures in research: a framework for food synergy. Am J Clin Nutr 78, 508S–513S. 12936941 10.1093/ajcn/78.3.508S

[11] Woolhead C, Gibney MJ, Walsh MC, (2015) A generic coding approach for the examination of meal patterns. Am J Clin Nutr 102, 316–323. 26085514 10.3945/ajcn.114.106112

[12] Hearty AP & Gibney MJ (2008) Analysis of meal patterns with the use of supervised data mining techniques – artificial neural networks and decision trees. Am J Clin Nutr 88, 1632–1642. 19064525 10.3945/ajcn.2008.26619

[13] de Oliveira Santos R, Fisberg RM, Lobo Marchioni DM, (2015) Dietary patterns for meals of Brazilian adults. Br J Nutr 114, 822–828. 26220554 10.1017/S0007114515002445

[14] Murakami K & Livingstone MB (2016) Energy density of meals and snacks in the British diet in relation to overall diet quality, body mass index, and waist circumference: findings from the National Diet and Nutrition Survey. Br J Nutr 116, 1479–1489. 27751190 10.1017/S0007114516003573

[15] Borgelt C (2012) Frequent item set mining. WIREs Data Mining Knowl Discov 2, 437–456.

[16] Murakami K, Livingstone MB & Sasaki S (2017) Establishment of a meal coding system for the characterization of meal-based dietary patterns in Japan. J Nutr 147, 2093–2101. 28904121 10.3945/jn.117.254896

[17] Murakami K, Sasaki S, Takahashi Y, (2008) Reproducibility and relative validity of dietary glycaemic index and load assessed with a self-administered diet-history questionnaire in Japanese adults. Br J Nutr 99, 639–648. 17764595 10.1017/S0007114507812086

[18] Katagiri R, Asakura K, Sasaki S, (2015) Estimation of habitual iodine intake in Japanese adults using 16 d diet records over four seasons with a newly developed food composition database for iodine. Br J Nutr 114, 624–634. 26197981 10.1017/S0007114515002019

[19] Tsugane S, Sasaki S, Kobayashi M, (2003) Validity and reproducibility of the self-administered food frequency questionnaire in the JPHC Study Cohort I: study design, conduct and participant profiles. J Epidemiol 13, S2–S12. 12701628 10.2188/jea.13.1sup_2 PMC9767693

[20] Sasaki S, Kobayashi M & Tsugane S (2003) Validity of a self-administered food frequency questionnaire used in the 5-year follow-up survey of the JPHC Study Cohort I: comparison with dietary records for food groups. J Epidemiol 13, S57–S63. 12701632 10.2188/jea.13.1sup_57 PMC9767694

[21] Council for Science and Technology (2005) Standard Tables of Food Composition in Japan, fifth revised and enlarged edition Tokyo, Japan: National Printing Bureau, 2005 (in Japanese).

[22] Ministry of Health, Labour and Welfare, Japan (2014) The National Health and Nutrition Survey in Japan, 2012. http://www.mhlw.go.jp/bunya/kenkou/eiyou/h24-houkoku.html (in Japanese).

[23] Northstone K, Ness AR, Emmett PM, (2008) Adjusting for energy intake in dietary pattern investigations using principal components analysis. Eur J Clin Nutr 62, 931–938. 17522611 10.1038/sj.ejcn.1602789 PMC2492394

[24] Tseng M, DeVellis RF, Maurer KR, (2000) Food intake patterns and gallbladder disease in Mexican Americans. Public Health Nutr 3, 233–243. 10948391 10.1017/s1368980000000276

[25] Okubo H, Murakami K, Sasaki S, (2010) Relative validity of dietary patterns derived from a self-administered diet history questionnaire using factor analysis among Japanese adults. Public Health Nutr 13, 1080–1089. 20074397 10.1017/S1368980009993211

[26] Okubo H, Sasaki S, Horiguchi H, (2006) Dietary patterns associated with bone mineral density in premenopausal Japanese farm women. Am J Clin Nutr 83, 1185–1192. 16685064 10.1093/ajcn/83.5.1185

[27] Uzhova I, Woolhead C, Timon CM, (2018) Generic meal patterns identified by latent class analysis: insights from NANS (National Adult Nutrition Survey). Nutrients 10, 310. 10.3390/nu10030310 PMC5872728 29509665

[28] Livingstone MB & Black AE (2003) Markers of the validity of reported energy intake. J Nutr 133, 895S–920S. 12612176 10.1093/jn/133.3.895S

[29] Livingstone MB (1995) Assessment of food intakes: are we measuring what people eat? Br J Biomed Sci 52, 58–67. 7549607

[30] Leech RM, Worsley A, Timperio A, (2015) Characterizing eating patterns: a comparison of eating occasion definitions. Am J Clin Nutr 102, 1229–1237. 26447152 10.3945/ajcn.115.114660

[31] Murakami K & Livingstone MB (2015) Eating frequency is positively associated with overweight and central obesity in US adults. J Nutr 145, 2715–2724. 26468490 10.3945/jn.115.219808

