# Supplementary material for: Applying a meal coding system to 16-d weighed dietary record data in the Japanese context: towards the development of simple meal-based dietary assessment tools
Source: J Nutr Sci. 2018 Nov 13;7:e29. doi: 10.1017/jns.2018.21 (PMC6238120; doi:10.1017/jns.2018.21)
Supplement: Supplementary file 1 [file S2048679018000216sup001.docx]

| **Supplementary Table S1.** Food groups used in the present study | |
| --- | --- |
| Food groups | Major food items |
| Rice | White rice |
| Bread | White bread, roll bread, croissant |
| Noodles | Japanese wheat noodle, spaghetti, buckwheat noodle, Chinese noodle,  instant noodle |
| Other grains | Wheat flour, panko, wheat products |
| Potatoes | Potato, konnyaku, yam, sweet potato, taro |
| Pulses* | Tofu, natto, other tofu products, beans |
| Vegetables | Onion, carrot, cabbage, radish, green onion, cucumber, Chinese cabbage,  lettuce, mushrooms, burdock, green pepper, spinach, tomato, pumpkin,  broccoli, leek, bean sprout, pickles, eggplant, lotus root, turnip, bamboo  short, wakame, hijiki |
| Fruit | Apple, persimmon, banana, satsuma, plum, pear, kiwi fruit, grape, blue berry |
| Fish† | Skipjack tuna, fish products, salmon, squid, sardine, shrimp, Pacific saury |
| Meat | Pork, chicken, beef, sausage, ham, ground meet, bacon |
| Eggs | Eggs |
| Dairy products | Milk (all varieties), cheese, yogurt (all varieties) |
| Confectioneries | Chocolate, candy, Japanese sweets, cake, doughnut, custard pudding, jelly,  biscuit, potato chips |
| Fruit and vegetable juice | 100% fruit juice, 100% vegetable juice |
| Alcoholic beverages | Sake, beer, shochu, wine |
| Soft drinks | Sugar-sweetened beverages |
| Tea and coffee (i.e., nonalcoholic and  noncaloric beverages) | Green tea, coffee, barley tea, black tea, oolong tea, brown rice tea |
| Sugars‡ | Jam, sugar |
| Fats and oils‡ | Vegetable oil, margarine, butter, mayonnaise |
| Seasonings‡ | Soy sauce, salt, miso |
| * Including nuts. |  |
| † Including shellfish. |  |
| ‡ Not considered in development of the substituted generic meal database. | |

| **Supplementary Table S2.** Meal codes and descriptions for the meal type “breakfast” and frequency of meals consumed* | | | | | | |
| --- | --- | --- | --- | --- | --- | --- |
| Meal code | | Food groups included | | Food groups not included | *n*^1^ | % |
| Vegetables and tea and coffee | | | | | | |
| 1101 | | Vegetables, tea and coffee, rice, pulses, fruit, dairy products | | --- | 129 | 3.4 |
| 1102 | | Vegetables, tea and coffee, rice, pulses, fruit | | Dairy products | 129 | 3.4 |
| 1103 | | Vegetables, tea and coffee, rice, pulses, eggs | | Fruit, fruit and vegetable juice, alcoholic beverages | 148 | 3.9 |
| 1104 | | Vegetables, tea and coffee, rice, pulses, fish | | Fruit, eggs, fruit and vegetable juice, alcoholic beverages, bread, noodles | 110 | 2.9 |
| 1105 | | Vegetables, tea and coffee, rice, pulses | | Fruit, eggs, fish, fruit and vegetable juice, alcoholic beverages | 153 | 4.0 |
| 1106 | | Vegetables, tea and coffee, rice, eggs | | Pulses | 268 | 7.1 |
| 1107 | | Vegetables, tea and coffee, rice, fish | | Pulses, eggs | 191 | 5.0 |
| 1108 | | Vegetables, tea and coffee, rice | | Pulses, eggs, fish, alcoholic beverages | 234 | 6.2 |
| 1109 | | Vegetables, tea and coffee, bread, dairy products, eggs | | Rice, noodles, alcoholic beverages | 203 | 5.4 |
| 1110 | | Vegetables, tea and coffee, bread, dairy products | | Rice, eggs, alcoholic beverages, soft drinks | 151 | 4.0 |
| 1111 | | Vegetables, tea and coffee, bread | | Rice, dairy products, alcoholic beverages, noodles, other grains | 98 | 2.6 |
| 1112 | | Vegetables, tea and coffee | | Rice, bread, alcoholic beverages | 116 | 3.1 |
| Bread and dairy products | | | | | | |
| 1201 | | Bread, dairy products, tea and coffee, fruit | | Vegetables, noodles, alcoholic beverages | 121 | 3.2 |
| 1202 | | Bread, dairy products, tea and coffee | | Fruit, vegetables, noodles, alcoholic beverages | 219 | 5.8 |
| 1203 | | Bread, dairy products, vegetables | | Tea and coffee, noodles, alcoholic beverages | 132 | 3.5 |
| 1204 | | Bread, dairy products | | Tea and coffee, vegetables, alcoholic beverages | 122 | 3.2 |
| Rice and vegetables | | | | | | |
| 1301 | | Rice, vegetables, dairy products | | Tea and coffee, bread | 161 | 4.3 |
| 1302 | | Rice, vegetables | | Tea and coffee, dairy products, alcoholic beverages | 170 | 4.5 |
| Rice and tea and coffee | | | | | | |
| 1401 | | Rice, tea and coffee | | Vegetables, alcoholic beverages | 204 | 5.4 |
| Bread and tea and coffee | | | | | | |
| 1501 | | Bread, tea and coffee | | Vegetables, dairy products, rice, noodles, alcoholic beverages | 156 | 4.1 |
| Dairy products and tea and coffee | | | | | | |
| 1601 | | Dairy products, tea and coffee | | Vegetables, rice, bread, noodles, alcoholic beverages | 134 | 3.5 |
| One food group | | | | | | |
| 1701 | | Tea and coffee only | | --- | 90 | 2.4 |
| All other combinations | | | | | | |
| 1801 | | All other combinations | | --- | 349 | 9.2 |
| Total | |  | |  | 3788 | 100 |
| * Total number of breakfasts consumed by 242 men and women. Food groups not listed may or may not be included in each breakfast, except for breakfasts coded as 1701. For the breakfast meal type (*n* = 3788), the most common combination of food groups consumed at >15 g was “vegetables and tea and coffee”. Within individual breakfasts consisting of some combination of “vegetables and tea and coffee” (meal code 1101-1112; *n* = 1930), the food group most commonly consumed was “rice”. Among all individual breakfasts which consisted of the “vegetables, tea and coffee, and rice” combination (meal code 1101-1108; *n* = 1362), the food group most commonly consumed was “pulses”. Within individual breakfasts which consisted of a “vegetables, tea and coffee, rice, and pulses” combination (meal code 1101-1105; *n* = 669), “fruit” was the most frequently consumed food item. Among all individual breakfasts which consisted of the “vegetables, tea and coffee, rice, pulses, and fruit” combination (meal code 1101-1102; *n* = 258), the food group most commonly consumed was “dairy products”. From this, the “vegetables, tea and coffee, rice, pulses, fruit, and dairy products” group (*n* = 129) was considered to be a food group combination pattern and was labeled with the generic meal code of 1101. The number of included food groups was arbitrarily determined based on the frequency of meals (breakfasts in this case) included in the code, taking account of the meaningfulness of food groups in characterizing the code as well as our intention that the total number of generic meals should not exceed 80. After completing the categorization of breakfasts which included combinations of “vegetables and tea and coffee”, the next most commonly consumed food group combination (“bread and dairy products” in this case; meal code 1201-1204) was identified and similarly categorized. This process was repeated stepwise until the next most commonly consumed food group combination represented <2% of the total breakfast number (specifically, “dairy products and tea and coffee”; meal code 1601). Breakfasts not only consisting of only one food group but also accounting for >2% of the number of total breakfasts were then identified as one category (“tea and coffee” in this case; meal code 1701). This process was repeated stepwise for each type of meal, with the result that a total of 80 generic meals accounting for all meal types were established. | | | | | | |
|  | | | | | | |
|  | | | | | | |
| **Supplementary Table S3.** Meal codes and descriptions for the meal type “lunch” and frequency of meals consumed* | | | | | | |
| Meal code | Food groups included | | | Food groups not included | *n*^1^ | % |
| Rice and vegetables | | | | | | |
| 2101 | Rice, vegetables, tea and coffee, fish, meat, eggs | | | --- | 143 | 3.7 |
| 2102 | Rice, vegetables, tea and coffee, fish, meat | | | Eggs | 232 | 6.1 |
| 2103 | Rice, vegetables, tea and coffee, fish, eggs | | | Meat | 213 | 5.6 |
| 2104 | Rice, vegetables, tea and coffee, fish, pulses | | | Meat, eggs, bread, soft drinks | 140 | 3.7 |
| 2105 | Rice, vegetables, tea and coffee, fish | | | Meat, eggs, pulses | 314 | 8.2 |
| 2106 | Rice, vegetables, tea and coffee, meat, eggs | | | Fish, alcoholic beverages | 203 | 5.3 |
| 2107 | Rice, vegetables, tea and coffee, meat, potatoes | | | Fish, eggs | 154 | 4.0 |
| 2108 | Rice, vegetables, tea and coffee, meat | | | Fish, eggs, potatoes | 293 | 7.7 |
| 2109 | Rice, vegetables, tea and coffee, pulses | | | Fish, meat, bread | 112 | 2.9 |
| 2110 | Rice, vegetables, tea and coffee | | | Fish, meat, pulses | 181 | 4.7 |
| 2111 | Rice, vegetables, meat, fish | | | Tea and coffee, bread | 102 | 2.7 |
| 2112 | Rice, vegetables, meat | | | Tea and coffee, fish | 210 | 5.5 |
| 2113 | Rice, vegetables, fish | | | Tea and coffee, meat | 179 | 4.7 |
| 2114 | Rice, vegetables | | | Tea and coffee, meat, fish, fruit and vegetable juice | 100 | 2.6 |
| Noodles and tea and coffee | | | | | | |
| 2201 | Noodles, tea and coffee, vegetables, meat | | | Rice | 140 | 3.7 |
| 2202 | Noodles, tea and coffee, vegetables | | | Meat, rice, fruit and vegetable juice | 152 | 4.0 |
| 2203 | Noodles, tea and coffee | | | Vegetables, bread, fruit and vegetable juice | 174 | 4.6 |
| Vegetables and meat | | | | | | |
| 2301 | Vegetables, meat | | | Rice | 196 | 5.1 |
| Rice and tea and coffee | | | | | | |
| 2401 | Rice, tea and coffee | | | Vegetables, noodles, alcoholic beverages | 151 | 3.9 |
| Bread and dairy products | | | | | | |
| 2501 | Bread, dairy products | | | Alcoholic beverages | 101 | 2.6 |
| All other combinations | | | | | | |
| 2601 | All other combinations | | | --- | 333 | 8.7 |
| Total |  | | |  | 3823 | 100 |
| * Total number of lunches consumed by 242 men and women. Food groups not listed may or may not be included in each lunch. For the lunch meal type (*n* = 3823), the most common combination of food groups consumed at >15 g was “rice and vegetables”. Within individual lunches consisting of some combination of “rice and vegetables” (meal code 2101-2114; *n* = 2576), the food group most commonly consumed was “tea and coffee”. Among all individual lunches which consisted of the “rice, vegetables, and tea and coffee” combination (meal code 2101-2110; *n* = 1985), the food group most commonly consumed was “fish”. Within individual lunches which consisted of a “rice, vegetables, tea and coffee, and fish” combination (meal code 2101-2105; *n* = 1042), “meat” was the most frequently consumed food item. Among all individual breakfasts which consisted of the “rice, vegetables, tea and coffee, fish, and meat” combination (meal code 2101-2102; *n* = 375), the food group most commonly consumed was “eggs”. From this, the “rice, vegetables, tea and coffee, fish, meat, and eggs” group (*n* = 143) was considered to be a food group combination pattern and was labeled with the generic meal code of 2101. The number of included food groups was arbitrarily determined based on the frequency of meals (lunches in this case) included in the code, taking account of the meaningfulness of food groups in characterizing the code as well as our intention that the total number of generic meals should not exceed 80. After completing the categorization of lunches which included combinations of “rice and vegetables”, the next most commonly consumed food group combination (“noodles and tea and coffee” in this case; meal code 2201-2203) was identified and similarly categorized. This process was repeated stepwise until the next most commonly consumed food group combination represented <2% of the total lunch number (specifically, “bread and dairy products”; meal code 2501). Lunches not only consisting of only one food group but also accounting for >1% of the number of total lunches were then identified as one category (none in this case). This process was repeated stepwise for each type of meal, with the result that a total of 80 generic meals accounting for all meal types were established. | | | | | | |
|  | | | | | | |
|  | | | | | | |
| **Supplementary Table S4.** Meal codes and descriptions for the meal type “dinner” and frequency of meals consumed* | | | | | | |
| Meal code | Food groups included | | | Food groups not included | *n*^1^ | % |
| Rice and vegetables | | | | | | |
| 3101 | Rice, vegetables, tea and coffee, fish, pulses, meat | | | --- | 166 | 4.3 |
| 3102 | Rice, vegetables, tea and coffee, fish, pulses, fruit | | | Meat, soft drinks | 100 | 2.6 |
| 3103 | Rice, vegetables, tea and coffee, fish, pulses | | | Meat, fruit | 238 | 6.2 |
| 3104 | Rice, vegetables, tea and coffee, fish, meat, potatoes | | | Pulses | 124 | 3.2 |
| 3105 | Rice, vegetables, tea and coffee, fish, meat | | | Pulses, potatoes | 184 | 4.8 |
| 3106 | Rice, vegetables, tea and coffee, fish, fruit | | | Pulses, meat, fruit and vegetable juice, soft drinks | 113 | 2.9 |
| 3107 | Rice, vegetables, tea and coffee, fish | | | Pulses, meat, fruit | 281 | 7.3 |
| 3108 | Rice, vegetables, tea and coffee, meat, potatoes | | | Fish, soft drinks | 242 | 6.3 |
| 3109 | Rice, vegetables, tea and coffee, meat, pulses | | | Fish, potatoes | 155 | 4.0 |
| 3110 | Rice, vegetables, tea and coffee, meat | | | Fish, pulses, potatoes | 231 | 6.0 |
| 3111 | Rice, vegetables, tea and coffee | | | Fish, meat, bread | 100 | 2.6 |
| 3112 | Rice, vegetables, fish, alcoholic beverages, meat | | | Tea and coffee, bread | 161 | 4.2 |
| 3113 | Rice, vegetables, fish, alcoholic beverages | | | Tea and coffee, meat | 165 | 4.3 |
| 3114 | Rice, vegetables, fish, pulses | | | Tea and coffee, alcoholic beverages | 157 | 4.1 |
| 3115 | Rice, vegetables, fish | | | Tea and coffee, pulses, alcoholic beverages | 173 | 4.5 |
| 3116 | Rice, vegetables, meat, potatoes | | | Tea and coffee, fish | 156 | 4.0 |
| 3117 | Rice, vegetables, meat | | | Tea and coffee, fish, potatoes, bread | 175 | 4.5 |
| 3118 | Rice, vegetables | | | Tea and coffee, fish, meat, soft drinks | 62 | 1.6 |
| Vegetables and meat | | | | | | |
| 3201 | Vegetables, meat, alcoholic beverages, fish | | | Rice, fruit and vegetable juice | 102 | 2.6 |
| 3202 | Vegetables, meat, alcoholic beverages | | | Rice, fish | 110 | 2.9 |
| 3203 | Vegetables, meat | | | Rice, alcoholic beverages | 188 | 4.9 |
| Vegetables and fish | | | | | | |
| 3301 | Vegetables, fish | | | Rice, meat | 172 | 4.5 |
| Rice and tea and coffee | | | | | | |
| 3401 | Rice, tea and coffee | | | Vegetables | 95 | 2.5 |
| All other combinations | | | | | | |
| 3501 | All other combinations | | | --- | 206 | 5.3 |
| Total |  | | |  | 3856 | 100 |
| * Total number of dinners consumed by 242 men and women. Food groups not listed may or may not be included in each dinner. For the dinner meal type (*n* = 3856), the most common combination of food groups consumed at >15 g was “rice and vegetables”. Within individual dinners consisting of some combination of “rice and vegetables” (meal code 3101-3118; *n* = 2983), the food group most commonly consumed was “tea and coffee”. Among all individual dinners which consisted of the “rice, vegetables, and tea and coffee” combination (meal code 3101-3111; *n* = 1934), the food group most commonly consumed was “fish”. Within individual dinners which consisted of a “rice, vegetables, tea and coffee, and fish” combination (meal code 3101-3107; *n* = 1206), “pulses” were the most frequently consumed food item. Among individual dinners which consisted of the “rice, vegetables, tea and coffee, fish, and pulses” combination (meal code 3101-3103; *n* = 504), the food group most commonly consumed was “meat”. From this, the “rice, vegetables, tea and coffee, fish, pulses, and meat” group (*n* = 166) was considered to be a food group combination pattern and was labeled with the generic meal code of 3101. The number of included food groups was arbitrarily determined based on the frequency of meals (dinners in this case) included in the code, taking account of the meaningfulness of food groups in characterizing the code as well as our intention that the total number of generic meals should not exceed 80. After completing the categorization of dinners which included combinations of “rice and vegetables”, the next most commonly consumed food group combination (“vegetables and meat” in this case; meal code 3201-3203) was identified and similarly categorized. This process was repeated stepwise until the next most commonly consumed food group combination represented <2% of the total dinner number (specifically, “rice and tea and coffee”; meal code 3401). Dinners not only consisting of only one food group but also accounting for >1% of the number of total dinners were then identified as one category (none in this case). This process was repeated stepwise for each type of meal, with the result that a total of 80 generic meals accounting for all meal types were established. | | | | | | |
|  | | | | | | |
|  | | | | | | |
|  | | | | | | |
|  | | | | | | |
|  | | | | | | |
| **Supplementary Table S5.** Meal codes and descriptions for the meal type “snack” and frequency of meals consumed* | | | | | | |
| Meal code | Food groups included | | Food groups not included | | *n*^1^ | % |
| Confectioneries and tea and coffee | | | | | | |
| 4101 | Confectioneries, tea and coffee, dairy products | | --- | | 324 | 9.9 |
| 4102 | Confectioneries, tea and coffee, fruit | | Dairy products | | 178 | 5.4 |
| 4103 | Confectioneries, tea and coffee | | Dairy products, fruit | | 616 | 18.9 |
| Dairy products and tea and coffee | | | | | | |
| 4201 | Dairy products, tea and coffee | | Confectioneries | | 332 | 10.2 |
| Fruit and tea and coffee | | | | | | |
| 4301 | Fruit, tea and coffee | | Confectioneries, dairy products | | 171 | 5.2 |
| Soft drinks and tea and coffee | | | | | | |
| 4401 | Soft drinks, tea and coffee | | Confectioneries, dairy products, fruit, other grains | | 113 | 3.5 |
| Confectioneries and dairy products | | | | | | |
| 4501 | Confectioneries, dairy products | | Tea and coffee, noodles, fish | | 76 | 2.3 |
| Alcoholic beverages and tea and coffee | | | | | | |
| 4601 | Alcoholic beverages, tea and coffee | | Confectioneries, dairy products, fruit, soft drinks, eggs, fruit and vegetable juice | | 68 | 2.1 |
| One food group | | | | | | |
| 4701 | Tea and coffee only | | --- | | 536 | 16.4 |
| 4702 | Confectioneries only | | --- | | 102 | 3.1 |
| 4703 | Soft drinks only | | --- | | 70 | 2.1 |
| All other combinations | | | | | | |
| 4801 | All other combinations | | --- | | 681 | 20.8 |
| Total |  | |  | | 3267 | 100 |
| * Total number of snacks consumed by 242 men and women. Food groups not listed may or may not be included in each snack, except for snacks coded as 4701, 4702, and 4703. For the snack meal type (*n* = 3267), the most common combination of food groups consumed at >15 g was “confectioneries and tea and coffee”. Within individual snacks consisting of some combination of “confectioneries and tea and coffee” (meal code 4101-4103; *n* = 1118), the food group most commonly consumed was “dairy products”. From this, the “confectioneries, tea and coffee, and dairy products” group (*n* = 324) was considered to be a food group combination pattern and was labeled with the generic meal code of 4101. The number of included food groups was arbitrarily determined based on the frequency of meals (snacks in this case) included in the code, taking account of the meaningfulness of food groups in characterizing the code as well as our intention that the total number of generic meals should not exceed 80. After completing the categorization of snacks which included combinations of “confectioneries and tea and coffee”, the next most commonly consumed food group combination (“dairy products and tea and coffee” in this case; meal code 4201) was identified and similarly categorized. This process was repeated stepwise until the next most commonly consumed food group combination represented <2% of the total snack number (specifically, “alcoholic beverages and nonalcoholic and noncaloric beverage”; meal code 4601). Snacks not only consisting of only one food group but also accounting for >2% of the number of total snacks were then identified as one category (meal code 4701-4703). This process was repeated stepwise for each type of meal, with the result that a total of 80 generic meals accounting for all meal types were established. | | | | | | |

| **Supplementary Table S6.** Crude food group intakes calculated using a standard food composition database and the substituted generic meal database and correlations between intakes calculated using the two databases (*n* 242)* | | | | | |
| --- | --- | --- | --- | --- | --- |
|  | Standard food composition database | | Generic meal database | | Spearman *r* |
|  | Mean | SD | Mean | SD |  |
| Rice (g/d) | 334.7 | 127.9 | 334.7 | 76.3 | 0.66 |
| Bread (g/d) | 35.2 | 26.6 | 35.2 | 22.7 | 0.83 |
| Noodles (g/d) | 72.3 | 42.9 | 72.3 | 30.1 | 0.59 |
| Other grains (g/d) | 26.2 | 23.1 | 26.2 | 6.2 | 0.30 |
| Potatoes (g/d) | 43.4 | 20.5 | 43.4 | 8.3 | 0.53 |
| Pulses (g/d)† | 63.7 | 35.8 | 63.7 | 15.9 | 0.66 |
| Vegetables (g/d) | 294.4 | 100.1 | 294.4 | 45.6 | 0.71 |
| Fruit (g/d) | 103.2 | 75.3 | 103.2 | 24.1 | 0.72 |
| Fish (g/d)‡ | 89.5 | 40.2 | 89.5 | 23.4 | 0.80 |
| Meat (g/d) | 73.7 | 35.0 | 73.7 | 17.4 | 0.79 |
| Eggs (g/d) | 40.1 | 16.0 | 40.1 | 8.9 | 0.61 |
| Dairy products (g/d) | 141.3 | 101.3 | 141.3 | 49.3 | 0.76 |
| Confectioneries (g/d) | 35.9 | 23.9 | 35.9 | 16.5 | 0.75 |
| Fruit and vegetable juice (g/d) | 13.9 | 32.3 | 13.9 | 5.4 | 0.33 |
| Alcoholic beverages (g/d) | 179.6 | 265.7 | 179.6 | 95.3 | 0.81 |
| Soft drinks (g/d) | 49.6 | 64.2 | 49.6 | 23.7 | 0.60 |
| Tea and coffee (g/d) | 705.8 | 305.9 | 705.8 | 179.4 | 0.70 |
| Sugar (g/d)§ | 10.9 | 10.8 | 10.9 | 2.2 | 0.44 |
| Fats and oils (g/d)§ | 13.9 | 5.8 | 13.9 | 1.6 | 0.49 |
| Seasonings (g/d)§ | 175.1 | 86.7 | 175.1 | 28.5 | 0.20 |
| * Resulting from the procedure used to develop the generic meal database, mean values of crude food group intakes (and energy intake) for the two calculations were theoretically identical. | | | | | |
| † Including nuts. | | | | | |
| ‡ Including shellfish. | | | | | |
| § Not considered in development of the substituted generic meal database. | | | | | |

| **Supplementary Table S7.** Crude nutrient intakes calculated using a standard food composition database and the substituted generic meal database and correlations between intakes calculated using the two databases (*n* 242)* | | | | | |
| --- | --- | --- | --- | --- | --- |
|  | Standard food composition database | | Generic meal database | | Spearman *r* |
|  | Mean | SD | Mean | SD |  |
| Protein (g/d) | 77.3 | 15.8 | 77.3 | 7.4 | 0.59 |
| Total fat (g/d) | 62.2 | 14.6 | 62.2 | 6.0 | 0.51 |
| SFA (g/d) | 17.4 | 4.8 | 17.4 | 2.1 | 0.60 |
| MUFA (g/d) | 22.2 | 6.0 | 22.2 | 2.2 | 0.55 |
| PUFA (g/d) | 14.1 | 3.1 | 14.1 | 1.2 | 0.33 |
| n-6 PUFA (g/d) | 11.4 | 2.6 | 11.4 | 1.0 | 0.33 |
| n-3 PUFA (g/d) | 2.7 | 0.7 | 2.7 | 0.4 | 0.55 |
| Marine-origin n-3 PUFA (g/d)† | 1.0 | 0.5 | 1.0 | 0.3 | 0.70 |
| EPA (g/d) | 0.3 | 0.2 | 0.3 | 0.1 | 0.70 |
| DHA (g/d) | 0.6 | 0.3 | 0.6 | 0.1 | 0.70 |
| α-linolenic acid (g/d) | 1.6 | 0.4 | 1.6 | 0.1 | 0.26 |
| Carbohydrate (g/d) | 283.2 | 61.6 | 283.2 | 26.3 | 0.45 |
| Alcohol (g/d) | 11.9 | 17.4 | 11.9 | 6.4 | 0.81 |
| Cholesterol (mg/d) | 364.1 | 98.1 | 364.1 | 51.3 | 0.60 |
| Total dietary fiber (g/d) | 15.3 | 4.3 | 15.3 | 1.8 | 0.67 |
| Soluble dietary fiber (g/d) | 3.3 | 0.9 | 3.3 | 0.4 | 0.59 |
| Insoluble dietary fiber (g/d) | 11.0 | 3.2 | 11.0 | 1.3 | 0.69 |
| Retinol (μg/d) | 383.9 | 405.5 | 383.9 | 66.2 | 0.30 |
| Vitamin A (retinol equivalent) (μg/d)‡ | 704.2 | 432.8 | 704.2 | 86.7 | 0.34 |
| α-Carotene (μg/d) | 495.9 | 250.0 | 495.9 | 79.1 | 0.29 |
| β-Carotene (μg/d) | 3319.0 | 1246.0 | 3319.0 | 519.4 | 0.50 |
| β-Carotene equivalent (μg/d)§ | 3816.0 | 1446.0 | 3816.0 | 580.8 | 0.48 |
| Cryptoxanthin (μg/d) | 350.8 | 325.2 | 350.8 | 93.6 | 0.49 |
| α-Tocopherol (mg/d) | 8.0 | 1.8 | 8.0 | 0.8 | 0.48 |
| Vitamin K (μg/d) | 247.4 | 104.2 | 247.4 | 46.6 | 0.66 |
| Thiamin (mg/d) | 1.0 | 0.2 | 1.0 | 0.1 | 0.47 |
| Riboflavin (mg/d) | 1.4 | 0.3 | 1.4 | 0.2 | 0.60 |
| Niacin (mg/d) | 19.7 | 5.5 | 19.7 | 2.1 | 0.56 |
| Vitamin B_6_ (mg/d) | 1.4 | 0.4 | 1.4 | 0.1 | 0.63 |
| Vitamin B_12_ (μg/d) | 8.6 | 3.8 | 8.6 | 1.6 | 0.64 |
| Folate (μg/d) | 382.3 | 115.7 | 382.3 | 47.0 | 0.63 |
| Pantothenic acid (mg/d) | 6.5 | 1.4 | 6.5 | 0.7 | 0.61 |
| Vitamin C (mg/d) | 116.7 | 43.1 | 116.7 | 16.2 | 0.66 |
| Sodium (mg/d) | 5155.0 | 1977.0 | 5155.0 | 508.5 | 0.32 |
| Potassium (mg/d) | 2845.0 | 668.2 | 2845.0 | 317.9 | 0.64 |
| Calcium (mg/d) | 584.3 | 177.2 | 584.3 | 86.5 | 0.74 |
| Magnesium (mg/d) | 302.2 | 75.1 | 302.2 | 33.7 | 0.58 |
| Phosphorus (mg/d) | 1168.0 | 251.8 | 1168.0 | 125.7 | 0.63 |
| Iron (mg/d) | 8.9 | 2.1 | 8.9 | 1.0 | 0.56 |
| Zinc (mg/d) | 9.0 | 1.9 | 9.0 | 0.9 | 0.48 |
| Copper (mg/d) | 1.3 | 0.3 | 1.3 | 0.2 | 0.63 |
| Manganese (mg/d) | 4.2 | 2.3 | 4.2 | 0.6 | 0.61 |
| * As a result of the procedure used to develop the generic meal database, mean values of crude nutrient intakes (and energy intake) for the two calculations were theoretically identical. | | | | | |
| † Sum of EPA, DPA, and DHA. | | | | | |
| ‡ Sum of retinol, 1/12 of β-carotene, 1/24 of α-carotene, and 1/24 of cryptoxanthin. | | | | | |
| § Sum of β-carotene, 1/2 of α-carotene, and 1/2 of cryptoxanthin. | | | | | |
|  | | | | | |

| **Supplementary Table S8.** Principal component (PC) analysis of meals based on 80 generic meal codes, showing the dominant loading values for each PC (6–11)* | | | | |
| --- | --- | --- | --- | --- |
| PC (variance) | Meal code | Meal type | Meal description | Loading value |
| 6 (3.3%) | 1105 | Breakfast | Vegetables, tea and coffee, rice, pulses (without fruit, eggs, fish, fruit and vegetable juice, alcoholic beverages) | -0.34 |
|  | 1106 | Breakfast | Vegetables, tea and coffee, rice, eggs (without pulses) | -0.29 |
|  | 1108 | Breakfast | Vegetables, tea and coffee, rice (without pulses, eggs, fish, alcoholic beverages) | -0.35 |
|  | 1203 | Breakfast | Bread, dairy products, vegetables (without tea and coffee, noodles, alcoholic beverages) | 0.51 |
|  | 1204 | Breakfast | Bread, dairy products (without tea and coffee, vegetables, alcoholic beverages) | 0.52 |
|  | 1801 | Breakfast | All other combinations | 0.38 |
|  | 2110 | Lunch | Rice, vegetables, tea and coffee (without fish, meat, pulses) | -0.30 |
|  | 4102 | Snack | Confectioneries, tea and coffee, fruit (without dairy products) | -0.31 |
|  | 4301 | Snack | Fruit, tea and coffee (without confectioneries, dairy products) | -0.55 |
|  | 4702 | Snack | Confectioneries only | 0.37 |
|  | 4801 | Snack | All other combinations | 0.37 |
| 7 (3.3%) | 1101 | Breakfast | Vegetables, tea and coffee, rice, pulses, fruit, dairy products | 0.52 |
|  | 1801 | Breakfast | All other combinations | -0.26 |
|  | 2201 | Lunch | Noodles, tea and coffee, vegetables, meat (without rice) | 0.59 |
|  | 2202 | Lunch | Noodles, tea and coffee, vegetables (without meat, rice, fruit and vegetable juice) | 0.61 |
|  | 3106 | Dinner | Rice, vegetables, tea and coffee, fish, fruit (without pulses, meat, fruit and vegetable juice, soft drinks) | 0.59 |
|  | 3107 | Dinner | Rice, vegetables, tea and coffee, fish (without pulses, meat, fruit) | 0.30 |
|  | 3112 | Dinner | Rice, vegetables, fish, alcoholic beverages, meat (without tea and coffee, bread) | -0.28 |
|  | 4703 | Snack | Soft drinks only | -0.27 |
| 8 (3.2%) | 1102 | Breakfast | Vegetables, tea and coffee, rice, pulses, fruit (without dairy products) | 0.57 |
|  | 2102 | Lunch | Rice, vegetables, tea and coffee, fish, meat (without eggs) | -0.32 |
|  | 2108 | Lunch | Rice, vegetables, tea and coffee, meat (without fish, eggs, potatoes) | -0.30 |
|  | 2301 | Lunch | Vegetables, meat (without rice) | 0.58 |
|  | 2501 | Lunch | Bread, dairy products (without alcoholic beverages) | 0.68 |
|  | 2601 | Lunch | All other combinations | 0.35 |
|  | 3108 | Dinner | Rice, vegetables, tea and coffee, meat, potatoes (without fish, soft drinks) | 0.36 |
|  | 3109 | Dinner | Rice, vegetables, tea and coffee, meat, pulses (without fish, potatoes) | 0.32 |
|  | 4103 | Snack | Confectioneries, tea and coffee (without dairy products, fruit) | 0.30 |
| 9 (3.1%) | 1103 | Breakfast | Vegetables, tea and coffee, rice, pulses, eggs (without fruit, fruit and vegetable juice, alcoholic beverages) | -0.33 |
|  | 1104 | Breakfast | Vegetables, tea and coffee, rice, pulses, fish (without fruit, eggs, fruit and vegetable juice, alcoholic beverages, bread, noodles) | -0.28 |
|  | 1106 | Breakfast | Vegetables, tea and coffee, rice, eggs (without pulses) | -0.32 |
|  | 1109 | Breakfast | Vegetables, tea and coffee, bread, dairy products, eggs (without rice, noodles, alcoholic beverages) | 0.45 |
|  | 1110 | Breakfast | Vegetables, tea and coffee, bread, dairy products (without rice, eggs, alcoholic beverages, soft drinks) | 0.53 |
|  | 1111 | Breakfast | Vegetables, tea and coffee, bread (without rice, dairy products, alcoholic beverages, noodles, other grains) | 0.30 |
|  | 2101 | Lunch | Rice, vegetables, tea and coffee, fish, meat, eggs | -0.37 |
|  | 2103 | Lunch | Rice, vegetables, tea and coffee, fish, eggs (without meat) | -0.43 |
|  | 2109 | Lunch | Rice, vegetables, tea and coffee, pulses (without fish, meat, bread) | 0.27 |
|  | 2110 | Lunch | Rice, vegetables, tea and coffee (without fish, meat, pulses) | 0.27 |
|  | 3101 | Dinner | Rice, vegetables, tea and coffee, fish, pulses, meat | -0.37 |
|  | 3104 | Dinner | Rice, vegetables, tea and coffee, fish, meat, potatoes (without pulses) | -0.34 |
|  | 3111 | Dinner | Rice, vegetables, tea and coffee (without fish, meat, bread) | 0.45 |
|  | 3113 | Dinner | Rice, vegetables, fish, alcoholic beverages (without tea and coffee, meat) | 0.29 |
|  | 4703 | Snack | Soft drinks only | -0.33 |
| 10 (3.0%) | 1104 | Breakfast | Vegetables, tea and coffee, rice, pulses, fish (without fruit, eggs, fruit and vegetable juice, alcoholic beverages, bread, noodles) | -0.38 |
|  | 1401 | Breakfast | Rice, tea and coffee (without vegetables, alcoholic beverages) | 0.48 |
|  | 2101 | Lunch | Rice, vegetables, tea and coffee, fish, meat, eggs | 0.30 |
|  | 4101 | Snack | Confectioneries, tea and coffee, dairy products | -0.39 |
|  | 4102 | Snack | Confectioneries, tea and coffee, fruit (without dairy products) | -0.31 |
|  | 4201 | Snack | Dairy products, tea and coffee (without confectioneries) | -0.33 |
|  | 4401 | Snack | Soft drinks, tea and coffee (without confectioneries, dairy products, fruit, other grains) | 0.56 |
|  | 4501 | Snack | Confectioneries, dairy products (without tea and coffee, noodles, fish) | -0.39 |
|  | 4701 | Snack | Tea and coffee only | 0.52 |
|  | 4703 | Snack | Soft drinks only | 0.35 |
| 11 (3.0%) | 1112 | Breakfast | Vegetables, tea and coffee (without rice, bread, alcoholic beverages) | 0.41 |
|  | 2103 | Lunch | Rice, vegetables, tea and coffee, fish, eggs (without meat) | 0.25 |
|  | 3103 | Dinner | Rice, vegetables, tea and coffee, fish, pulses (without meat, fruit) | 0.28 |
|  | 3112 | Dinner | Rice, vegetables, fish, alcoholic beverages, meat (without tea and coffee, bread) | -0.59 |
|  | 3113 | Dinner | Rice, vegetables, fish, alcoholic beverages (without tea and coffee, meat) | -0.42 |
|  | 3116 | Dinner | Rice, vegetables, meat, potatoes (without tea and coffee, fish) | -0.41 |
|  | 3117 | Dinner | Rice, vegetables, meat (without tea and coffee, fish, potatoes, bread) | -0.32 |
|  | 3203 | Dinner | Vegetables, meat (without rice, alcoholic beverages) | 0.65 |
|  | 3501 | Dinner | All other combinations | 0.25 |
| * Loading values were calculated from PC analysis using the percentage contribution of daily energy intake of the 80 generic meals for each participant (*n* = 242). Only loading values <-0.25 or >0.25 are displayed. The results on other PCs (1–5) are shown in Table 5. | | | | |
|  |  |  |  |  |
